# Cerebrospinal Fluid MicroRNA Signatures as Diagnostic Biomarkers in Brain Tumors

**DOI:** 10.3390/cancers11101546

**Published:** 2019-10-12

**Authors:** Alena Kopkova, Jiri Sana, Tana Machackova, Marek Vecera, Lenka Radova, Karolina Trachtova, Vaclav Vybihal, Martin Smrcka, Tomas Kazda, Ondrej Slaby, Pavel Fadrus

**Affiliations:** 1 Central European Institute of Technology (CEITEC), Masaryk University, Brno 625 00, Czech Republic; alena.kopkova@ceitec.muni.cz (A.K.); 375588@mail.muni.cz (T.M.); marek.vecera@ceitec.muni.cz (M.V.); 232848@mail.muni.cz (L.R.); trachtova@mail.muni.cz (K.T.); 2 Department of Comprehensive Cancer Care, Masaryk Memorial Cancer Institute, Brno 656 53, Czech Republic; 3 Department of Neurosurgery, University Hospital Brno, Brno 625 00, Czech Republic; Vybihal.Vaclav@fnbrno.cz (V.V.); Smrcka.Martin@fnbrno.cz (M.S.); 4 Faculty of Medicine, Masaryk University, Brno 625 00, Czech Republic; tomas.kazda@mou.cz; 5 Department of Radiation Oncology, Masaryk Memorial Cancer Institute, Faculty of Medicine, Masaryk University, Brno 656 53, Czech Republic

**Keywords:** glioblastoma, meningioma, brain metastases, microRNA, cerebrospinal fluid

## Abstract

Central nervous system (CNS) malignancies include primary tumors that originate within the CNS as well as secondary tumors that develop as a result of metastatic spread. Circulating microRNAs (miRNAs) were found in almost all human body fluids including cerebrospinal fluid (CSF), and they seem to be highly stable and resistant to even extreme conditions. The overall aim of our study was to identify specific CSF miRNA patterns that could differentiate among brain tumors. These new biomarkers could potentially aid borderline or uncertain imaging results onto diagnosis of CNS malignancies, avoiding most invasive procedures such as stereotactic biopsy or biopsy. In total, 175 brain tumor patients (glioblastomas, low-grade gliomas, meningiomas and brain metastases), and 40 non-tumor patients with hydrocephalus as controls were included in this prospective monocentric study. Firstly, we performed high-throughput miRNA profiling (Illumina small RNA sequencing) on a discovery cohort of 70 patients and 19 controls and identified specific miRNA signatures of all brain tumor types tested. Secondly, validation of 9 candidate miRNAs was carried out on an independent cohort of 105 brain tumor patients and 21 controls using qRT-PCR. Based on the successful results of validation and various combination patterns of only 5 miRNA levels (miR-30e, miR-140, let-7b, mR-10a and miR-21-3p) we proposed CSF-diagnostic scores for each tumor type which enabled to distinguish them from healthy donors and other tumor types tested. In addition to this primary diagnostic tool, we described the prognostic potential of the combination of miR-10b and miR-196b levels in CSF of glioblastoma patients. In conclusion, we performed the largest study so far focused on CSF miRNA profiling in patients with brain tumors, and we believe that this new class of biomarkers have a strong potential as a diagnostic and prognostic tool in these patients.

## 1. Introduction

Malignancies of the central nervous system (CNS) consist of primary tumors and secondary tumors that originate in different parts of a body and occur in CNS as brain metastasis. These two groups of CNS tumors count almost 40 patients per 100,000 persons worldwide and the incidence rate is still growing. The main types of primary brain tumors include gliomas, ependymomas and meningiomas [1,2]. Gliomas originate from glial cells and are classified by histopathological and molecular features into four classes, more generally into low grade gliomas (LGG, WHO I and II), and high-grade gliomas (HGG, WHO III and IV), when the most common glioma is glioblastoma multiforme (GBM) [3]. GBM, with the incidence rate of 4.7–5.7 cases per every 100,000, is also one of the most aggressive brain tumors, and even after therapy, the median survival time is only around 14.6 months [4]. On the other hand, meningiomas are in most cases slowly growing tumors and represent most common adult primary brain tumors, characterized by almost two times more frequent occurrence in females than in males. According to the World Health Organization (WHO) classification they are divided into three grades (grade I, grade II, also referred as atypical meningioma and grade III). The majority of grade I is benign and counts almost 80% of all meningiomas. Compared to a good prognosis of grade I, atypical meningiomas grow and progress more rapidly and represent about 15%. Meningiomas grade III are rarer and they occur around 2% and show the most aggressive behaviour [5,6,7]. Brain metastases are also one of the most frequently occurring brain malignancies with poor overall survival [8].

Following the fact that prognosis and therapy depends on detecting the brain tumor type early and accurate diagnosis is crucial, this could significantly affect life quality as well as survival of the patients. Current diagnosis approaches are based on imaging methods such as computed tomography (CT) and magnetic resonance (MRI) with subsequent histological examination of biopsy. Nevertheless, these approaches are limited by brain tumor localization and heterogeneity. Therefore, it is still necessary to look for diagnostic approaches and biomarkers that are at the same time robust, sensitive and specific, and whose collection is not very invasive. The use of biomarkers found in body fluids (liquid biomarkers) appears to be a suitable approach for detecting a variety of pathological conditions including cancer. Cerebrospinal fluid (CSF), which bathes all the CNS and is in direct contact with any possible pathological components, is considered as the ideal source of these biomarkers for detecting brain tumors [9,10]. 

MicroRNAs (miRNAs) are single stranded, non-coding RNA which are 18–25 nucleotides in length, and post transcriptionally regulate gene expression. These molecules are usually tissue specific and involved in the pathogenesis of many diseases [11]. Circulating miRNAs were found in almost all human body fluids including CSF and they seem to be highly stable and resist extreme conditions [12] Moreover, several studies have shown that deregulated levels of CSF miRNAs are associated with malignant tumors of CNS [13,14,15]. Taken together, analysis of miRNAs in CSF of brain tumor patients might help to develop a new diagnostic platform enabling more precise diagnostic approaches. 

## 2. Material and Methods

### 2.1. Collection of Clinical Samples and CSF Processing and Storage

CSF samples were collected from the Department of Neurosurgery, University Hospital Brno, Czech Republic. Informed consent approved by the local Ethical Committee of University Hospital Brno (ethic code: 14-08-27-01) on 27 August, 2014, was obtained from each patient before the lumbar puncture. In the discovery phase, 89 CSF samples taken from 32 glioblastoma, 14 low-grade glioma, 11 meningioma, 13 brain metastasis patients, and 19 non-tumor patients were used for small RNAseq analysis. Subsequently, 126 CSF samples were used for the validation phase (41 glioblastoma, 8 low-grade glioma, 44 meningioma, 12 metastasis patients and 21 non-tumor patients) (summarized in Table 1). Briefly, 4–6 mL of CSF samples were obtained during the lumbar puncture between the L3 and L5 vertebrae before surgical intervention in brain tumor patients or during standard therapy management of patients with normal-pressure hydrocephalus (non-tumor patients). CSF samples containing blood-derived cells were excluded. Subsequently, CSF samples were centrifuged at 500× *g* for 10 min at 4 °C (Eppendorf 5810 R, Hamburg, Germany), and the supernatant were aliquoted to 1 mL tubes and stored at −80 °C. The sample processing took no more than one hour. In glioblastoma patients we also collected follow-up clinical data and information on overall survival (OS).

### 2.2. RNA Isolation

Urine microRNA Purification Kit (Norgen Biotek, Thorold, ON, Canada) was used for isolations of all CSF samples according to manufacturer‘s protocol with few modifications: (i) at the elution step, samples were incubated for 20 mins on the column, (ii) we decreased the volume of elution solution to 20 µL, (iii) elution step was repeated twice with the same sample.

### 2.3. Small RNA Sequencing

Library preparation was performed by CleanTag Library preparation kit (Trilink Biotechnologies, L-3206, San Diego, CA, USA) according to manufacturer’s protocol. The maximum volume of RNA sample was always added to reaction. Libraries were purified by Agencourt AMPure XP (Beckman Coulter, Brea, CA, USA). The sequencing analysis was performed by Next 500/550 High Output v2 Kit with 75 cycles using the NextSeq 500 instrument (both Illumina, San Diego, CA, USA). For miRNA mapping and analysis, an online tool Chimira (Enright Lab at EMBL-EBI, Cambridge, UK) was used. Obtained data were subsequently statistically evaluated in the environment of statistical language R using the Bioconductor edgeR and DESeq2 package.

### 2.4. cDNA Synthesis and qRT-PCR

In the validation phase of the study, cDNA synthesis was performed by TaqMan^TM^ Advanced miRNA cDNA Synthesis kit followed by qRT-PCR using TaqMan^TM^ Fast Advanced Master Mix with individual TaqMan Advanced miRNA assays (all ThermoFisher Scientific, Waltham, MA, USA) on the QuantStudio™ 3D Digital PCR Instrument (ThermoFisher Scientific, Waltham, MA, USA). All reactions were held according to manufacturer’s protocol.

### 2.5. Data Analysis

All real-time PCR reactions were run in triplicates and average threshold cycle and SD values were calculated. 2^-ΔCt^ method (ΔCt = Ct(miRNA) − Ct(average(let-7i-5p, miR-151a-3p, miR-423-3p)) was used for Ct values normalization. Reference miRNAs let-7i-5p, miR-151a-3p, and miR-423-3p were chosen based on the analysis of small RNAseq data using algorithms geNorm and NormFinder. LogFC was calculated as logarithm of ratio between specific miRNA average expressions of two statistically compared groups. All analyses (Mann-Whitney non-parametric tests, ROC analyses, Kaplan-Meier and long-rank test) were performed using GraphPad Prism version 6.00 (GraphPad Software, San Diego, CA, USA). *p*-values of <0.05 were considered statistically significant.

For discovery of diagnostic signatures (DS), and prognostic miRNA combination, logistic regression was performed. Successfully validated miRNAs were introduced into a bidirectional stepwise logistic regression model and the final model was taken as that which maximizes the Akaike information criterion. Formulas for calculation of Diagnostic Scores (DS):

Brain tumors DS = −1.742 + (miR-30e × 1.139) + (miR-140 × −2.320);

Glioblastoma DS = −2.876 + (let-7b × −1.823) + (miR-21-3p × 4.380) + (miR-10a × 2.244);

Meningioma DS = 2.472 + (let-7b × −0.064) + (miR-21-3p × −10.826) + (miR-10a × −1.278);

Brain metastasis DS = −2.571 + (let-7b × 1.746) + (miR-21-3p × 11.672) + (miR-10a × −1.114).

## 3. Results

**Figure 1:**
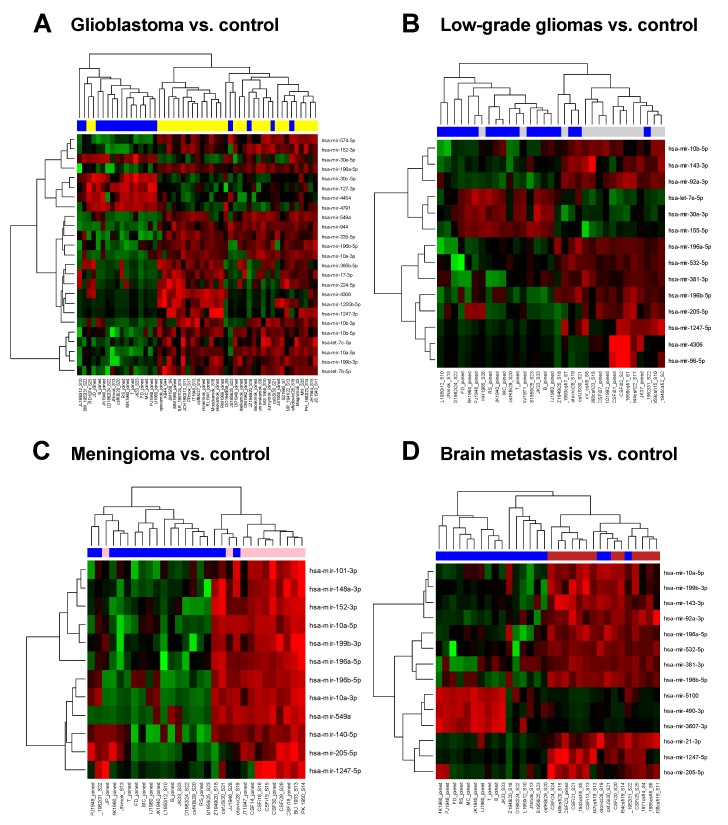
Hierarchical clustering based on cerebrospinal fluid (CSF) miRNA expression profiles of glioblastomas and controls (**A**); low-grade gliomas and controls; (**B**) meningiomas and controls (**C**); and brain metastases and controls (**D**). Blue color always indicates CSF specimen collected from control individual. A gradient of green and red colors is used in the heatmap (green color indicates lower expression whereas red color indicates higher expression of individual miRNAs in analyzed samples).

**Figure 2:**
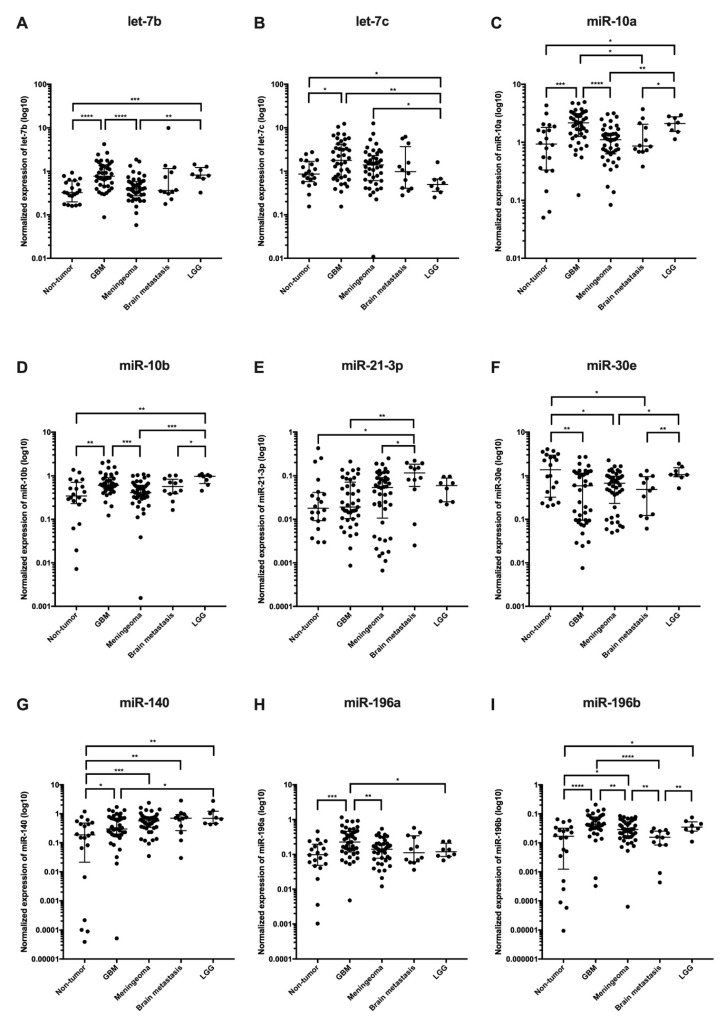
Validation of candidate cerebrospinal fluid miRNA biomarkers (**A** let-7b, **B** let-7c, **C** miR-10a, **D** miR-10b, **E** miR-21-3p, **F** miR-30e, **G** miR-140, **H** miR-196a, **I** miR-196b). In controls, patients with glioblastoma (GBM), meningioma, brain metastasis, and low-grade glioma (LGG).

**Figure 3:**
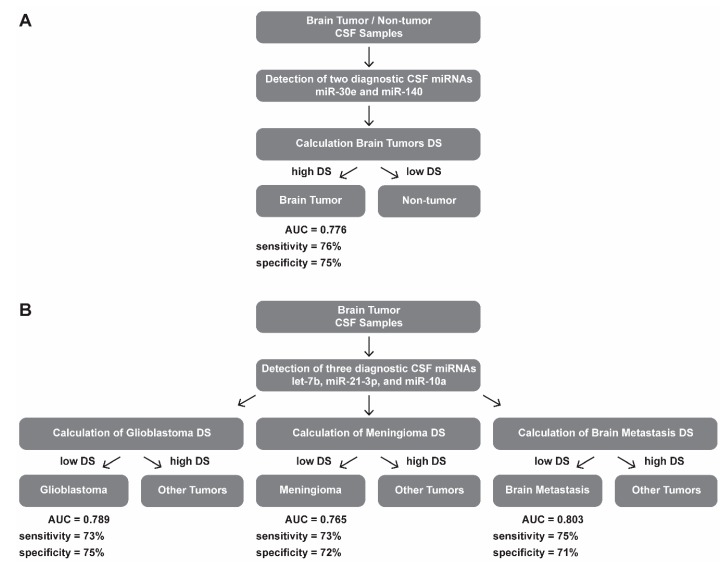
Diagnostic schemas for brain tumor patients stratification of (**A**) brain tumor and non-tumor patients; and (**B**) glioblastoma, meningioma and brain metastasis patients based on detection of selected miRNAs in CSF. DS = Diagnostic Score; AUC = Area Under Curve; and CSF = Cerebrospinal fluid.

**Figure 4:**
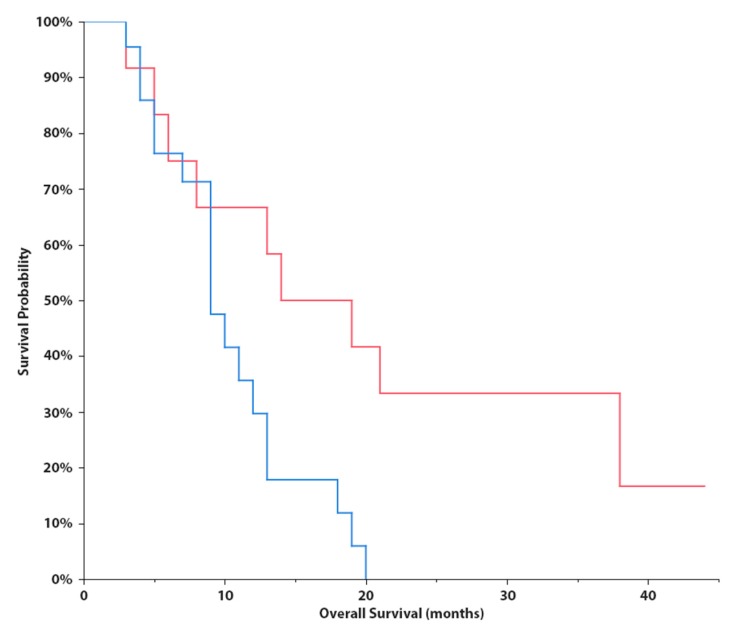
Kaplan-Meier survival curves estimating overall survival in patients with glioblastoma according to combined cerebrospinal fluid levels of miR-10b and miR-196b (low levels in red - median OS = 16.5 months; and high levels in blue - median OS = 9 months; p = 0.0170, Log-Rank test).

In the discovery phase of the study, we successfully performed small RNA sequencing of 89 CSF samples collected from patients with brain tumors and hydrocephalus (non-tumor controls). When CSF miRNA profiles from glioblastoma patients were compared to CSF from controls, we identified 25 miRNAs to be significantly deregulated (*p* < 0.001, Table 2, Figure 1A). Low-grade glioma miRNA profiles significantly differed from controls in levels of 14 miRNA (*p* < 0.1, Table 3, Figure 1B). In CSF of meningioma and brain metastasis patients 12 miRNAs (*p* < 0.01) and 14 miRNAs (*p* < 0.001) identified to differentially expressed, respectively (Table 4 and Table 5, Figure 1C–D). Based on the fold-change, significance specificity, and uniqueness for various tumor types, we selected 9 miRNAs (let-7a, let-7b, miR-10a, miR-10b, miR-21-3p, miR-30e, miR-140, miR-196a and miR-196b) to be validated in CSF specimens of independent groups of patients (41 GBMs, 8 low-grade gliomas, 44 meningiomas, 12 brain metastases and 21 non-tumor patients). Results of the validation phase are shown in Figure 2. We also proposed the Diagnostic Scores (DS) for each tumor type and the schema for stratification of brain tumor and non-tumor patients (Figure 3A), and glioblastoma, meningioma and brain metastasis patients (Figure 3B), based on a detection of miR-30e and miR-140, and let-7b, miR-21-3p and miR-10a in CSF respectively. Through ROC analysis we identified the DS thresholds enabling to stratify patients with the highest sensitivity and specificity. Specifically, DS threshold −1.883 was calculated based on CSF levels of miR-30e, and miR-140 enabled stratification of brain tumor patients and non-cancer donors with the sensitivity 76% and specificity 75% (Figure 3A). DS thresholds −0.525, 0.033 and −2.164 were calculated based on CSF levels of let-7b, miR-21-3p and miR-10a enable stratify GBM (sensitivity 73% and specificity 75%), meningioma (sensitivity 73% and specificity 72%) and brain metastasis (sensitivity 75% and specificity 71%) from other brain tumor types (Figure 3B). In addition to this primary diagnostic approach, we described prognostic potential of the combination of miR-10b and miR-196b levels in CSF of glioblastoma patients (Figure 4). Whereas the median overall survival (OS) in patients with miR-10b/miR-196b high levels was 9 months, in patients with low levels the median OS was 16.5 months.

## 4. Discussion

The overall aim of our study was to identify specific CSF miRNA patterns that could differentiate among brain tumors in the largest cohort of patients published so far. From a translational perspective, our aim was to identify new biomarkers that can aid borderline or uncertain imaging results onto the diagnosis of CNS malignancies, avoiding most invasive procedures such as stereotactic biopsy or biopsy. Therapeutic strategies could be planned in advance improving patients’ quality of life. Moreover, the identification of such biomarkers could help in finding alternative therapeutic targets. Based on the knowledge that CSF is the CNS biological fluid, that it flows only in the CNS, and it is easily collectable by a spinal tap at the lumbar cisternae level, we also hypothesized that CSF would be the ideal biological fluid to find CNS biomarkers [15]. On the other hand, miRNAs have demonstrated their great ability to classify human cancers [16,17] and to be very stable RNAs in CSF [9]. CSF also has the advantage to contain fewer miRNAs than blood plasma or serum, which are, instead, flowing throughout the body and, thus, less tissue specific and more vulnerable to contaminations from blood cellular components. 

Our results indicate a very good potential of CSF miRNAs in primary diagnostics of brain tumors and their potential supportive value in the diagnostic process in cases with borderline or uncertain imaging results. We identified CSF miRNA signatures for all studied cancer types. Some of the miRNAs identified in our study were already described by others, for instance miR-10b increased in CSF of GBM patients [14] or miR-21 in CSF of patients with brain metastasis [14,15]. Until now, there was no study published which focused on miRNA levels in CSF of meningioma patients. 

In the validation phase of our study, we confirmed very good reproducibility and robustness of CSF miRNAs as biomarkers. We successfully validated all miRNAs identified by small RNA sequencing to have significantly (adj. *p* < 0.05) different levels in CSF of glioblastoma cases also by use of qRT-PCR method. In cases of meningioma we confirmed 2 out of 5 miRNAs, and in brain metastasis 2 out of 6 miRNAs were independently validated. We suppose that lower validation success in meningiomas and brain metastasis is caused by smaller cohorts in both explorative and validation phases in comparison with GBM. Last but not least, we successfully validated 4 out of 6 miRNAs in low-grade gliomas with different levels (*p* < 0.05) in the exploratory phase. We used these miRNAs to establish a diagnostic schema for brain tumor patient stratification based on the detection of only five miRNAs in CSF. Moreover, we were able to show that 2 miRNAs measured in pre-operatively collected CSF indicated prognostic functioning in patients with glioblastoma (Figure 4). This could potentially present clinically very important information since in cases with borderline resectable tumors, prognostic information could also be considered as a factor for the decision making process regarding surgical intervention. We also believe that another potential clinical application of our observations is an improvement of the low-grade glioma diagnosis since occurrence of glioblastoma CSF miRNA profiles in these cases could be considered an indicator of the presence of high-grade focuses which could be “overlooked” within the standard diagnostic process. Specifically, our results indicate that let-7c, miR-140 and miR-196a show significantly different levels in glioblastoma and low-grade glioma patients’ CSF. Although a lot of studies have described possible functions of miRNAs at the cellular and molecular levels, there are only a few studies focused on the cell-free miRNAs to be brain tumor biomarkers, and none from them describe our successfully validated miRNAs as potentially diagnostic biomarkers. Only Regazzo et al., detected let-7c levels in pre-surgery blood serum obtained from GBM, WHO II-III glioma and meningioma patients and healthy donors. They did not observe any differences among examined groups, so this corresponds with previous conclusions that CSF seems to be a more sensitive diagnostic biofluid in comparison with blood plasma and serum in brain tumors [18,19]. Many more studies have been published in relation to tumor brain biomarkers and tissue miRNAs. Among all, miR-196a and miR-196b showed increased expression levels in GBMs relative to both anaplastic astrocytomas and normal brain tissues, which is consistent with our results since both miRNAs had significantly higher levels in CSFs from GBM patients than from non-tumor donors. Moreover, miR-196a seems to be associated with glioma progression and the prognostic role of miR-196b was suggested in GBM patients [20]. Another two studies described increasing tissue expression levels of miR-196a upon progression of low-grade gliomas to the GBM [21,22]. In accordance with our results, miR-10b was upregulated in GBM tissue compared to brain tissue of non-neoplastic controls. However, we did not observe different CSF levels of miR-10b between GBM and WHO I-III gliomas like Visani et al. [23]. Whereas our study shows significantly higher levels of let-7b in CSF from GBM patients in comparison to non-tumor donors, in GBM tissues the levels were described to have lower expression of let-7b [24] indicating active release of this tumor suppressive miRNA by glioblastoma cells. Similarly, miR-140 showed increased expression upon progression of WHO grade II to glioblastomas [25] whereas our results indicate higher CSF levels of miR-140 in low-grade glioma patients. However, analysis of these small non-coding RNAs in CSF is still not fully standardized and there are many factors that could bias the results. Thus, optimization and standardization of individual steps of the whole analytical process could bring CSF miRNAs closer to the clinical utilization [26].

## 5. Conclusions

In conclusion, we performed the largest study so far focused on CSF miRNA profiling in patients with brain tumors. We described significant differences in CSF miRNA levels in patients with all tested tumor types by the use of small RNA sequencing which is the most comprehensive method of miRNA profiling. The majority of the miRNA candidates we have also successfully validated in independent cohorts of patients by standard qRT-PCR method. Based on our results, we believe that CSF miRNAs have a strong potential as the diagnostic and prognostic biomarkers in patients with brain tumors.

## Figures and Tables

**Table 1:** Groups of patients included in this study.

Group	Discovery Cohort	Validation Cohort
	*N* = 89	*N* = 126
controls (hydrocephalus)	19	21
glioblastoma	32	41
low-grade glioma	14	8
meningiomas	11	44
brain metastasis	13	12

**Table 2:** MicroRNAs with the most significantly different levels in cerebrospinal fluid of glioblastoma patients in comparison to controls (*p* < 0.001) supplemented with additional miRNAs tested in the validation phase of the study listed at the bottom of the table (in italics). All miRNAs selected for the validation phase are in bold; and logFC = binary logarithm of Fold Change.

Genes	logFC	Average Expression	*p*-Value	Adjusted *p*-Value
**miR-196a-5p**	**4.22**	**9.76**	**<0.00001**	**<0.00001**
miR-4306	3.99	1.75	<0.00001	<0.00001
**miR-10a-5p**	**2.64**	**14.78**	**<0.00001**	**<0.00001**
miR-4791	−4.01	3.37	<0.00001	<0.00001
miR-30c-5p	−2.20	7.87	<0.00001	<0.00001
miR-1255b-5p	3.18	1.41	<0.00001	<0.00001
**miR-30e-5p**	**−1.21**	**10.67**	**<0.00001**	**<0.00001**
miR-549a	4.06	3.71	<0.00001	<0.00001
**miR-10b-5p**	**2.21**	**16.15**	**<0.00001**	**<0.00001**
**miR-196b-5p**	**3.76**	**5.32**	**<0.00001**	**<0.00001**
miR-199b-3p	1.53	14.09	<0.00001	<0.00001
miR-127-3p	−1.79	7.98	<0.00001	0.00027
**let-7b-5p**	**1.13**	**17.84**	**<0.00001**	**0.00011**
miR-574-5p	1.45	10.21	<0.00001	0.00027
miR-152-3p	1.39	10.42	<0.00001	0.00027
miR-1247-3p	2.43	0.43	<0.00001	0.00033
miR-944	3.55	3.33	<0.00001	0.00033
**let-7c-5p**	**1.22**	**15.75**	**<0.00001**	**0.00022**
miR-224-5p	3.05	2.21	<0.00001	0.00039
miR-4454	−3.39	5.10	<0.00001	0.00039
miR-335-5p	3.38	4.92	<0.00001	0.00039
miR-17-3p	2.84	2.29	<0.00001	0.00039
miR-365b-5p	2.96	1.62	<0.00001	0.00059
miR-10b-3p	3.10	2.45	<0.00001	0.00072
miR-10a-3p	3.71	4.25	<0.00001	0.00078
***miR-140-5p***	***1.89***	***4.77***	***0.01080***	***0.05594***
***miR-21-3p***	***1.14***	***1.97***	***0.11732***	***0.27104***

**Table 3:** MicroRNAs with the most significantly different levels in cerebrospinal fluid of low grade glioma patients in comparison to controls (*p* < 0.01) supplemented with additional miRNAs tested in the validation phase of the study listed at the bottom of the table (in italics). All miRNAs selected for the validation phase are in bold; and logFC = binary logarithm of Fold Change.

Genes	logFC	Average Expression	*p*-Value	Adjusted *p*-Value
miR-381-3p	3.37	5.97	0.00000	0.00334
miR-205-5p	4.31	4.29	0.00011	0.03741
miR-92a-3p	1.88	13.27	0.00013	0.03741
miR-532-5p	2.28	8.05	0.00031	0.05360
miR-1247-5p	2.72	1.55	0.00032	0.05360
miR-155-5p	−2.15	8.63	0.00049	0.06435
**miR-196a-5p**	**2.45**	**9.76**	**0.00054**	**0.06435**
**miR-196b-5p**	**2.94**	**5.32**	**0.00087**	**0.07357**
miR-96-5p	1.79	−0.24	0.00148	0.09360
miR-4306	2.55	1.75	0.00157	0.09360
**miR-30e-3p**	**−1.36**	**10.03**	**0.00139**	**0.09360**
miR-143-3p	1.27	14.69	0.00088	0.07357
hsa-let-7e-5p	−0.87	11.75	0.00143	0.09360
**miR-10b-5p**	**1.58**	**16.15**	**0.00088**	**0.07357**
***let-7b-5p***	***0.25***	***17.84***	***0.36461***	***0.77936***
***let-7c-5p***	***−0.06***	***15.75***	***0.86022***	***0.95823***
***miR-10a-5p***	***1.46***	***14.78***	***0.00520***	***0.14588***
***miR-140-5p***	***2.24***	***4.77***	***0.00914***	***0.20606***
***miR-21-3p***	***1.00***	***1.97***	***0.27474***	***0.73439***

**Table 4:** MicroRNAs with the most significantly different levels in cerebrospinal fluid of meningioma patients in comparison to controls (*p* < 0.001) supplemented with additional miRNAs tested in the validation phase of the study listed at the bottom of the table (in italics). All miRNAs selected for the validation phase are in bold; and logFC = binary logarithm of Fold Change.

Genes	logFC	Average Expression	*p*-Value	Adjusted *p*-Value
**miR-196a-5p**	**4.01**	**9.76**	**<0.00001**	**0.00001**
**miR-10a-5p**	**2.82**	**14.78**	**<0.00001**	**0.00003**
miR-549a	4.69	3.71	<0.00001	0.00059
**miR-196b-5p**	**4.06**	**5.32**	**<0.00001**	**0.00103**
miR-199b-3p	1.65	14.09	0.00001	0.00109
miR-101-3p	1.56	11.93	0.00001	0.00118
miR-152-3p	1.62	10.42	0.00001	0.00137
miR-10a-3p	4.37	4.25	0.00003	0.00315
miR-148a-3p	1.70	11.92	0.00005	0.00489
**miR-140-5p**	**3.39**	**4.77**	**0.00008**	**0.00695**
miR-1247-5p	3.26	1.55	0.00011	0.00841
miR-205-5p	4.45	4.29	0.00014	0.00953
***miR-10b-5p***	***1.77***	***16.15***	***0.00039***	***0.01893***
***let-7b-5p***	***0.69***	***17.84***	***0.01600***	***0.13214***
***let-7c-5p***	***0.83***	***15.57***	***0.00830***	***0.09333***
***miR-30e-5p***	**−*0.05***	***10.67***	***0.84957***	***0.95825***
***miR-21-3p***	***0.75***	***1.97***	***0.44158***	***0.76889***

**Table 5:** MicroRNAs with the most significantly different levels in cerebrospinal fluid of brain metastases patients in comparison to controls (*p* < 0.001) supplemented with additional miRNAs tested in the validation phase of the study listed at the bottom of the table (in italics). All miRNAs selected for the validation phase are in bold; and logFC = binary logarithm of Fold Change.

Genes	logFC	Average Expression	*p*-Value	Adjusted *p*-Value
miR-5100	−5.28	2.33	<0.00001	0.00011
miR-92a-3p	2.50	13.27	<0.00001	0.00011
miR-143-3p	1.86	14.69	<0.00001	0.00023
**miR-196a-5p**	**3.34**	**9.76**	**<0.00001**	**0.00033**
**miR-196b-5p**	**4.08**	**5.32**	**<0.00001**	**0.00033**
miR-490-3p	−4.82	1.37	<0.00001	0.00036
miR-1247-5p	3.70	1.55	<0.00001	0.00036
miR-199b-3p	1.67	14.09	<0.00001	0.00036
**miR-21-3p**	**4.22**	**1.97**	**0.00001**	**0.00062**
miR-3607-3p	−4.21	0.79	0.00001	0.00067
miR-205-5p	4.87	4.29	0.00001	0.00067
miR-532-5p	2.77	8.05	0.00001	0.00067
miR-381-3p	3.24	5.97	0.00001	0.00067
**miR-10a-5p**	**2.28**	**14.78**	**0.00001**	**0.00067**
***miR-10b-5p***	***1.72***	***16.15***	***0.00041***	***0.00919***
***let-7b-5p***	***0.43***	***17.84***	***0.11602***	***0.38548***
***let-7c-5p***	***0.39***	***15.75***	***0.21442***	***0.51985***
***miR-140-5p***	***2.60***	***4.77***	***0.00247***	***0.03024***
***miR-30e-5p***	***−0.47***	***10.67***	***0.08626***	***0.32384***
